# Phenotypic and molecular assessment of antimicrobial resistance profile of airborne *Staphylococcus* spp. isolated from flats in Kraków

**DOI:** 10.1007/s10453-017-9481-7

**Published:** 2017-04-10

**Authors:** Anna Lenart-Boroń, Katarzyna Wolny-Koładka, Katarzyna Juraszek, Andrzej Kasprowicz

**Affiliations:** 1 Department of Microbiology, University of Agriculture, Mickiewicza Ave. 24/28, 31-059 Kraków, Poland; 2 Faculty of Biotechnology and Horticulture, University of Agriculture, 29 Listopada Ave 54, 31-425 Kraków, Poland; 3 Centre for Microbiological Research and Autovaccines, Sławkowska 17, 31-016 Kraków, Poland

**Keywords:** Antibiotics, Bacteria, Molecular characterization, Polymerase chain reaction (PCR), Resistance

## Abstract

Bacteria of the genus *Staphylococcus* were isolated from air sampled from living spaces in Kraków (Poland). In total, 55 strains belonging to the genus *Staphylococcus* were isolated from 45 sites, and 13 species of coagulase-negative staphylococci were identified. The species composition of studied airborne microbiota contains *Staphylococcus* species that are rarely infectious to humans. Most commonly isolated species comprised *S. hominis* and *S. warneri*. The disk-diffusion tests showed that the collected isolates were most frequently resistant to erythromycin. The PCR technique was employed to search for genes conferring the resistance in staphylococci to antibiotics from the group of macrolides, lincosamides and streptogramins. The analyzed *Staphylococcus* isolates possessed simultaneously 4 different resistance genes. The molecular analysis with the use of specific primers allowed to determine the most prevalent gene which is *mphC,* responsible for the resistance to macrolides and for the enzymatic inactivation of the drug by phosphotransferase. The second most often detected gene was *msrA1,* which confers the resistance of staphylococci to macrolides and is responsible for active pumping of antimicrobial particles out of bacterial cells.

## Introduction

Nowadays, people spend increasingly more time indoors but these places often do not meet the indoor air quality standards. Indoor environment is frequently conquered by pathogenic and drug-resistant microorganisms, hazardous to the health and life of residents (Górny and Dutkiewicz 2002). The major source of airborne microbiological contamination in living spaces is usually people emitting microorganisms during processes such as talking, coughing, sneezing and desquamation. Another important factor affecting the composition of airborne microflora is the process of microbial migration from the outdoor environment, which is of a particular importance during summer and autumn when buildings are naturally ventilated (Butarewicz 2005). Significant internal sources of bioaerosols include also pets, whose hair may constitute a factor of pathogen transmission (Jo and Kang 2006). Pathogenic microorganisms in flats may also dwell in surface linings and construction materials, such as wallpaper, insulating materials, plasterboards and many others. Microbiological air pollution is also favored by the increasing presence of air conditioners, fans and heating systems that are ideal place for dwelling, proliferation and subsequent spreading of microorganisms (Gąska-Jędruch and Dudzińska 2009).

Among bioaerosol-forming bacteria, special attention should be paid to the genus *Staphylococcus*, which colonizes humans and animals. Staphylococci colonize mainly skin, epithelia and mucous membranes, in particular moist areas of nose, armpits and in groins (Irving et al. 2008). Bacteria from the genus *Staphylococcus* can survive for a long period of time in the air, easily being transferred and contaminating the environment. Within this genus, the most important etiological factors of human infections are *S. aureus, S. epidermidis*, *S. saprophyticus* (Bartoszewicz-Potyrała and Przondo-Mordarska 2002) and *S. haemolyticus* (Botelho et al. 2012), which are often found in indoor air (Botelho et al. 2012; Hospodsky et al. 2012). Majority of current bioaerosol research is focused on the microbiological analysis of the air quality, taking into consideration only the total number of bacteria and, based on that, assigning different classes of air purity. Also the assessment of antimicrobial resistance of aerosol-forming bacteria which may pose epidemiological risk is often neglected.

Today, the most frequently identified mechanisms of antimicrobial resistance in the genus *Staphylococcus* are: enzymatic inactivation of antibiotic molecules involving hydrolases and transferases; changing affinity for a drug or duplication of functions of the target site as a result of mutation within the gene encoding the protein being the target site for an antimicrobial agent (which may cause reduction in its binding affinity) and active removal of an antimicrobial agent from the cell with the so-called “efflux mechanism” (CDC 2002). The most common mechanism of resistance to the antibiotics analyzed within this study, i.e., macrolides, lincosamides and streptogramins, involves posttranscriptional modification of ribosomal 23S rRNA as a result of the activity of adenino-N6-methyltransferase. Structural genes encoding methyltransferase in different bacterial species are most commonly called *erm* (*erythromycin ribosome methylation*). Modification of 23S rRNA by methyltransferases causes the formation of multiple resistance in bacteria, as it inhibits three groups of antibiotics—macrolides, lincosamides and group B streptogramins (MLS_B_). This type of resistance may be encoded on plasmids or bacterial chromosomes, e.g., by a *Staphylococcus aureus* plasmid pE194, whose resistance may be either constitutive or inducible (Leclerq 2002). Another type of macrolide resistance is based on enzymatic inactivation of antibiotics, which can result from cleavage of macrolide lactone rings by esterase, or modification of its structure by enzymes from the group of transferases and by phosphorylase and hydrolase. These mechanisms confer the bacterial resistance only to one from three classes of the MLS group of antibiotics, e.g., genes *vat* or *vatB* in staphylococci encode acetyltransferase inactivating group A streptogramins (Stogios et al. 2014). Bacterial resistance to erythromycin is mainly dependent on the ability of antibiotics to overcome the bacterial cell envelope barriers, which is based on passive diffusion. Bacteria that are naturally resistant to antibiotics are characterized by high susceptibility to macrolides. The concentration of macrolides inside the cell of a susceptible *Staphylococcus* strain is a hundred times greater than in the naturally resistant bacteria. Erythromycin is a weak base, and its ionization decreases with increasing pH, and its non-ionic form much easier penetrates the outer membrane. If the antibiotic reaches the interior of the cell, it is possible that a third type of resistance mechanism is revealed, which involves its active removal from the cell. Bacterial genes involved in this process include, e.g., *msrA, msrB* or *vga*. These genes encode families of ABC transporters (multiprotein transport systems, ATP-binding cassettes) (Chesneau et al. 2005; Markiewicz and Kwiatkowski 2012).

This research was aimed at isolation of airborne staphylococci from living spaces, their species identification, phenotypic evaluation of their resistance to the selected groups of antimicrobial agents and detection of some of the resistance-conferring genes. The obtained results should help to determine the possible contamination of living spaces by pathogenic species of the *Staphylococcus* genus, and to assess—in the case of infection—whether the antibiotic administration could be effective in decreasing adverse health outcome appearance among exposed individuals.

## Material and methods

### Air sampling

Bacteria from the genus *Staphylococcus* were isolated from the air of 45 randomly chosen flats in Kraków. The flats were selected based on their location (city center), floor area (between 30 and 60) and the number of residents (between 2 and 4). Air sampling was conducted twice per year—in spring (mid-April 2015) and winter (mid-January 2016). The measurements were taken in triplicates using MAS-100 air sampler (Merck) on working days in the afternoons (when the residents were present). Each time 100 L of air was sampled within 1 min, in accordance with the requirements specified in the standard PN-Z-04008-08:1989 (1989). The air sampler was placed 1.5 m above the floor (one sampling site per each flat) in the center of the living room. Chapman medium (Biocorp, Poland) was used in order to isolate bacteria from the genus *Staphylococcus*. After sampling, the Petri plates were immediately transported to the laboratory where they were incubated at 36 ± 1 °C for 48 h.

### Isolation of pure cultures of staphylococci

Species identification of the collected *Staphylococcus* isolates was carried out according to the methodology given by Kloos and Schleifer (1975), Kloos and Bannerman (1994), Gaillot et al. (2000), Murray (2007) and Lenart-Boroń et al. (2016). Pure cultures of *Staphylococcus* spp. were obtained by streaking of macroscopically characterized colonies which grew on the Chapman medium. Subsequently, Gram-stained smears were microscopically observed, and the furazolidone susceptibility test was performed in order to discriminate the *Staphylococcus* genus from the *Micrococcus* strains. Furazolidone-susceptible strains were classified as *Staphylococcus* spp., while the resistant ones—as *Micrococcus* spp. Then, the catalase test was conducted to exclude the presence of catalase-negative streptococci in the analyzed material. Next, using free coagulase, the differentiation within the genus *Staphylococcus* was made to separate coagulase-positive and coagulase-negative species. The final stage of phenotypic identification was biochemical tests conducted using the API Staph system (bioMerieux).

### Antimicrobial resistance, disk-diffusion method

In order to investigate the susceptibility of the *Staphylococcus* spp. isolates to antibiotics most commonly used in treatment, disk-diffusion tests were conducted on Mueller–Hinton Agar II (bioMerieux) with the use of the following antimicrobial agents: cefoxitin (FOX 30 µg), ciprofloxacin (CIP 5 µg), erythromycin (E 15 µg), gentamycin (CN 10 µg), clindamycin (DA 2 µg), tetracycline (TE 30 µg) and trimethoprim/sulfamethoxazole (SXT 1.25/23.75 µg). The susceptibility evaluation was performed according to the guidelines of the National Reference Centre for Antimicrobial Susceptibility (KORLD), contained in the “Recommendations for the selection of tests for susceptibility and chemotherapeutics testing. Determination of susceptibility of Gram–positive cocci from the genus *Staphylocococcus* 2010” (Żabicka and Hryniewicz 2010).

Methicillin-susceptible *S. aureus* ATCC 25923 and methicillin-resistant *S. aureus* MR reference strains from the collection of the Jan Bober Centre of Microbiological Research and Autovaccines in Kraków were used as controls, and the results were interpreted according to the recommendations of KORLD and EUCAST (Hryniewicz 2014).

### Determination of genes conferring the resistance to MLS group antibiotics

DNA extraction from pure cultures of staphylococci was performed using the Genomic Mini kit (A&A Biotechnology, Poland), following the instructions provided by the manufacturer. Then, PCR amplification with specific primers (Table 1) was used to search for 17 genes conferring different mechanisms of resistance in bacteria from the genus *Staphylococcus* to antibiotics from groups of macrolides, lincosamides and streptogramins. The reactions were performed in a T100 thermal cycler (BioRad, USA), under temperature conditions optimal for the respective primers (Table 1). All PCRs were performed in a volume of 25 μL containing 50 ng of DNA template, 12.5 pM of each primer, 2.5 mM of dNTP, 1 × PCR buffer and 1 U DreamTaq DNA polymerase (ThermoScientific, USA).

**Table 1:** PCR primers and reaction conditions

No	Gene	Primer	Sequence (5′-3′)	PCR conditions	Product length (bp)	Reference
1	*msr(A)*	msrA-1	GGCACAATAAGAGTGTTTAAAGG AAGTTATATCATGAATAGATTGTCCTGTT	25 (1 min 94 °C; 1 min 50 °C; 90 s 72 °C)	940	Lina et al. (1999)
		msrA-2				
2	*msr(A1)*	msrA1-1	GCAAATGGTGTAGGTAAGACAACT ATCATGTGATGTAAACAAAAT	30 (30 s 95 °C; 45 s 50 °C; 2 min 72 °C)	400	Sutcliffe et al. (1996)
		msrA1-2				
3	*msr(B)*	msrB-1	TATGATATCCATAATAATTATCCAATC AAGTTATATCATGAATAGATTGTCCTGTT	25 (1 min 94 °C; 1 min 50 °C; 90 s 72 °C)	595	Schmitz et al. (2000)
		msrB-2				
4	*ere(A)*	ereA-1	AACACCCTGAACCCAAGGGACG CTTCACATCCGGATTCGCTCGA	30 (30 s 95 °C; 45 s 50 °C; 2 min 72 °C)	420	Lui et al. (2009)
		ereA-2				
5	*ere(B)*	ereB-1	AGAAATGGAGGTTCATACTTACCA CATATAATCATCACCAATGGCA	30 (30 s 95 °C; 45 s 50 °C; 2 min 72 °C)	546	Sutcliffe et al. (1996)
		ereB-2				
6	*mph(A)*	mphA-1	AACTGTACGCACTTGC GGTACTCTTCGTTACC	30 (30 s 95 °C; 45 s 50 °C; 2 min 72 °C)	837	Sutcliffe et al. (1996), Lui et al. (2009)
		mphA-2				
7	*mph(C)*	mphC-1	GAGACTACCAAGAAGACCTGACG CATACGCCGATTCTCCTGAT	30 (30 s 94 °C; 30 s 59 °C; 1 min 72 °C)	722	Luthje and Schwarz (2006)
		mphC-2				
8	*lnu(A)*	lnuA-1	GGTGGCTGGGGGGTAGATGTATTAACTGG GCTTCTTTTGAAATACATGGTATTTTTCGATC	30 (30 s 94 °C; 30 s 57 °C; 1 min 72 °C)	323	Lina et al. (1999)
		lnuA-2				
9	*erm(A)*	ermA-1	TCTAAAAAGCATGTAAAAGAA CTTCGATAGTTTATTAATATTAGT	30 (30 s 95 °C; 45 s 52 °C; 2 min 72 °C)	645	Sutcliffe et al. (1996), Lui et al. (2009)
		ermA-2				
10	*erm(A1)*	ermA1-1	GTTCAAGAACAATCAATACAGAG GGATCAGGAAAAGGACATTTTAC	30 (30 s 94 °C; 30 s 52 °C; 1 min 72 °C)	421	Lina et al. (1999)
		ermA1-2				
11	*erm(B)*	ermB-1	GAAAAGGTACTCAACCAAATA AGTAACGGTACTTAAATTGTTTAC	30 (30 s 95 °C; 45 s 52 °C; 2 min 72 °C)	639	Sutcliffe et al. (1996), Lui et al. (2009)
		ermB-2				
12	*erm(B1)*	ermB1-1	CCGTTTACGAAATTGGAACAGGTAAAGGGC GAATCGAGACTTGAGTGTGC	30 (30 s 94 °C; 30 s 55 °C; 1 min 72 °C)	359	Lina et al. (1999), Lui et al. (2009)
		ermB1-2				
13	*erm(C)*	ermC-1	TCAAAACATAATATAGATAAA GCTAATATTGTTTAAATCGTCAAT	30 (30 s 95 °C; 45 s 52 °C; 2 min 72 °C)	642	Sutcliffe et al. (1996), Lui et al. (2009)
		ermC-2				
14	*vga*	vga-1	CCAGAACTGCTATTAGCAGATGAA AAGTTCGTTTCTCTTTTCGACG	30 (30 s 94 °C; 30 s 54 °C; 1 min 72 °C)	470	Lina et al. (1999)
		vga-2				
15	*vgb*	vgb-1	ACTAACCAAGATACAGGACC TTATTGCTTGTCAGCCTTCC	30(1 min 94 °C; 1 min 53 °C;2 min 72 °C)	734	Lina et al. (1999)
		vgb-2				
16	*vat*	vat-1	CAATGACCATGGACCTGATC CTTCAGCATTTCGATATCTC	30 (30 s 94 °C; 30 s 52 °C; 1 min 72 °C)	619	Lina et al. (1999)
		vat-2				
17	*vat(B)*	vatB-1	CCCTGATCCAAATAGCATATATCC CTAAATCAGAGCTACAAAGT	30 (30 s 94 °C; 30 s 52 °C; 1 min 72 °C)	602	Lina et al. (1999)
		vatB-2				

The products were visualized by electrophoresis in 1% agarose gel in TBE buffer, stained with SimplySafe (EurX, Poland). Bands were analyzed on a UV transilluminator (BioRad, USA) using the size marker GeneRuler TM DNA Ladder Mix, 100–1000 bp, (ThermoScientific, USA).

## Results

Two species: *S. hominis* and *S. warneri* prevailed among collected isolates (Table 2). *Staphylococcus aureus*, which is potentially most dangerous pathogenic staphylococcal species, was absent in the studied material. A total of 55 strains of coagulase-negative *Staphylococcus* spp., belonging to 13 species, were isolated from the air of 45 randomly selected flats in Kraków.

**Table 2:** Share of individual staphylococci species in the isolated material

No	Species	Number of isolates/percentage
1	*S. hominis*	10/18
2	*S. warneri*	9/16
3	*S. cohnii* subsp. *cohnii*	7/13
4	*S. sciuri*	6/11
5	*S. haemolyticus*	4/8
6	*S. epidermidis*	3/5
7	*S. saprophyticus*	3/5
8	*S. xylosus*	3/5
9	*S. auricularis*	3/5
10	*S. capitis*	2/4
11	*S. lugdunensis*	2/4
12	*S. lentus*	2/4
13	*S. simulans*	1/2
Total		55/100

Disk-diffusion tests allowed to determine the antimicrobial resistance profile of the isolated *Staphylococcus* spp. strains (Table 3). The tested strains were most frequently resistant to erythromycin and least frequently to ciprofloxacin and gentamycin.

**Table 3:** Antimicrobial resistance of the studied *Staphylococcus* spp. strains

Antimicrobial agent	Number/percentage of resistant strains (*n* = 55)
erythromycin	35/63.6
tetracycline	13/23.6
clindamycin	11/20
cefoxitin	4/7.3
trimethoprim/sulphamethoxazole	3/5.5
ciprofloxacin	2/3.6
gentamycin	2/3.6

Among the analyzed staphylococci, 17 isolates (30.9%) were susceptible to all tested antimicrobial agents, 20 isolates (36.3%) were resistant to one, 10 isolates (18.2%) to 2, 6 isolates (10.9%) to 3, 1 strain (1.8%) was resistant to 5 and yet another 1 strain to 6 antimicrobial agents tested in this study.

The incidence of the mechanisms of resistance to macrolide, lincosamide and streptogramin antibiotics is shown in Table 4.

**Table 4:** Number/percentage of strains resistant to MLS_B_ antibiotics

Mechanism of resistance	Number/percentage of strains (*n* = 55)
MS_B_	21/38.2
Susceptible	19/34.5
MLS_B_ constitutive	8/14.5
MLS_B_ inducible	5/9.1
Error in determination or L phenotype	2/3.6

Table 5 shows the results of PCR amplifications demonstrating the presence of genes conferring the resistance to MLS antibiotics. The data revealed that most of the analyzed *Staphylococcus* spp. isolates possessed *mph(C)* gene which confers the resistance to macrolides and is responsible for enzymatic inactivation of antibiotics by phosphotransferase. The second most frequent gene was *msr(A1),* which confers the resistance to macrolides in staphylococci (MS_B_). Genes *erm(B), msr(A), msr(B), lnu(A), erm, erm(A), ere(A), ere(B), erm(A1), vga* were present in less than half of the tested strains. On the other hand, the genes *mph(A), erm(B1), vgb, vat, vat(B)* were not found.

**Table 5:** Prevalence of drug resistance genes in the tested strains

Gene	Number/percentage of strains (*n* = 55)	Resistance to antibiotics
*mphC*	50/91	Macrolides
*msrA1*	41/75	Macrolides
*ermB*	24/44	MLS
*msrA*	18/32	MLS
*msrB*	18/32	Macrolides
*lnuA*	17/31	Lincosamides
*ermC*	9/16	MLS
*ermA*	3/5	MLS
*ereA*	2/4	Macrolides
*ereB*	1/2	Macrolides
*ermA1*	1/2	MLS
*vga*	1/2	Streptogramins A
*mphA*	0	Macrolides
*ermB1*	0	MLS
*vgb*	0	Streptogramins A i B
*vat*	0	Streptogramins A
*vatB*	0	Streptogramins A

*MLS* macrolides, lincosamides and streptogramins B

Among the tested isolates, one—identified as *S. warneri*—had as many as 7 resistance-determining genes, such as: *msr(A), msr(A1), msr(B), mph(C), lnu(A), erm(B), erm(C).* In contrast, another strain also belonging to the same species did not have any of the analyzed genes. Most of the isolates, i.e., 17, had 4 genes conferring the resistance to MLS group of antibiotics, while further 12 strains had 2 different resistance genes. Table 6 summarizes the presented data concerning the species determination of airborne staphylococci, their antimicrobial resistance and the resistance-determining genes.

**Table 6:** Summary of antimicrobial resistance and the resistance-determining genes in the airborne staphylococci analyzed in this study

No	Species	Antimicrobial resistance (no. of strains with detected mechanism)*	Resistance-determining genes (no. of strains with detected genes)
1.	*S. hominis*	E (2); DA (2); TE (2); CIP (1); FOX (1); CN (1); MLS_B_ constitutive (2)	*mph(C)* (8); *msr(A)* (1); *msr(B)* (1*); msr(A1)* (5); *ere(A)* (1); *erm(B)* (5); *erm(C)* (1); *lnu(A)* (2);
2.	*S. warneri*	E (7); DA (1); TE (4); MS_B_ (3); MLS_B_ constitutive (1); MLS_B_ inducible (3)	*msr(A)* (5); *msr(A1)* (7); *msr(B)* (4); *mph(C)* (8); *lnu(A)* (4); *erm(B)* (3); *erm(C)* (2)
3.	*S. cohnii* subsp. *cohnii*	E (6); DA (1); MS_B_ (5); MLS_B_ constitutive (1)	*msr(A)* (3); *msr(A1)* (6); *msr(B)* (3*); mph(C)* (7); *lnu(A)* (3); *erm(A)* (1); *erm(B)* (3*); erm(C)* (1)
4.	*S. sciuri*	E (4); DA (2); TE (2); CIP (1); MS_B_ (1); MLS_B_ constitutive (1); MLS_B_ inducible (2); error in determination or L phenotype (1)	*msr(A)* (1*); msr(B)* (1); *msr(A1)* (4*); mph(C)* (6); *lnu(A)* (3); *erm(B)* (4); *erm(C)* (2)
5.	*S. haemolyticus*	E (3); DA (1); MS_B_ (2); MLS_B_ constitutive (1)	*msr(A)* (2); *msr(A1*) (3); *msr(B)* (2); *mph(C)* (4); *erm(C)* (1)
*6.*	*S. epidermidis*	E (3); TE (2); SXT (1); MS_B_ (3)	*msr(A)* (1); *msr(A1)* (3); *msr(B)* (1); *ere(A)* (1); *mph(C)* (3)
7.	*S. saprophyticus*	E (2); DA (1); MS_B_ (1)	*msr(A1)* (1); *mph(C)* (2); *erm(B)* (2); *erm(C)* (1)
8.	*S. xylosus*	E (3); DA (1); TE (1); MS_B_ (2); MLS_B_ constitutive (1)	*msr(A)* (2); *msr(A1)* (3); *msr(B)* (2*); mph(C)* (3); *lnu(A)* (1)*; erm(A)* (1); *erm(A1)* (1); *erm(B)* (1); *erm(C)* (1)
9.	*S. auricularis*	E (2); DA (1); TE (1); FOX (1); CN (1); MS_B_ (1); MLS_B_ constitutive (1)	*msr(A)* (3); *msr(A1)* (3); *msr(B)* (3); *mph(C)* (2)
10.	*S. capitis*	E (1); DA (1); TE (1); SXT (1); MS_B_ (1); error in determination or L phenotype (1)	*msr(A1)* (2); *ere(B)* (1); *mph(C)* (2); *lnu(A)* (1); *erm(B)* (1)
11.	*S. lugdunensis*	S	*msr(A1)* (1); *mph(C)* (2); *lnu(A)* (1); *erm(B)* (2)
12.	*S. lentus*	E (2); SXT (1); FOX (2); MS_B_ (2)	*msr(A1)* (2); *mph(C)* (2); *lnu(A)* (2); *erm(A)* (1); *erm(B)* (2)
13.	*S. simulans*	S	*msr(A1)*; *msr(B)*; *mph(C)*; *erm(B)*; *vga*

* E—erythromycin; DA—clindamycin; TE—tetracycline; FOX—cefoxitin; CN—gentamycin; CIP—ciprofloxacin; SXT—trimethoprim/sulfamethoxazole; MS_B_—strains resistant to macrolides and streptogramins B; MLS_B_ constitutive—strains showing constitutive type of resistance to macrolides, lincosamides and streptogramins B; MLS_B_ inducible—strains showing inducible type of resistance to macrolides, lincosamides and streptogramins B

## Discussion

The results of our study indicate that most of airborne staphylococci isolated from the studied flats possibly poses no threat to their healthy residents. Nevertheless, the presence of potentially pathogenic strains—although in small numbers—can cause infections particularly in elderly, children and in immunocompromised people (Gąska-Jędruch and Dudzińska 2009). The most numerous species in the analyzed material was *S. hominis* (18%), which is usually isolated from human skin armpits, arms, legs and trunk. *S. hominis* usually does not cause serious infections in humans, but it is an opportunistic pathogen that can contribute to infections of hospitalized patients weakened by long-term pharmacological treatment (Jiang et al. 2012). On the other hand, studies conducted by Pastuszka et al. (2000) in flats of Upper Silesia (Poland) showed that the main bacterial species isolated from indoor air was *S. epidermidis*, which was present in 76% of studied flats constituting 14% of the total amount of bacteria. In our study, *S. epidermidis* isolates constituted only 5% of all *Staphylococcus* species. The second most frequently isolated species in the presented research was *S. warneri* (16%), which is a component of the human epithelium and mucous membrane microflora, but in some cases can be the cause of bacteriemia in long-term hospitalized patients (Campoccia et al. 2010). Another species among the identified staphylococci was *S. cohnii* subsp. *cohnii* (13%), which may cause pneumonia, acute cholecystitis, endocarditis, bacteriemia and urinary tract infections. *S. cohnii* subsp. *cohnii* is mostly isolated in hospitals (Hu et al. 2014). The last most frequently observed staphylococci were the species defined as the one resistant to novobiocin—*S. sciuri* (11%). It is regarded as a typical animal-related species, isolated from skin and mucous membranes, as well as from food products of animal origin. In humans, it can be present in nasopharynx, on skin and in urogenital system. Clinicians are increasingly interested in this species, as it has been recognized as one of the etiological factors of diseases such as endocarditis, peritonitis, septic shock, urinary tract infections and endophthalmitis (Dakić et al. 2005).

Most of the analyzed strains were resistant to erythromycin (63.6%) and tetracycline (23.6%), and least frequently to ciprofloxacin and gentamycin (3.6% for both antimicrobials). Such large number of strains resistant to erythromycin may result from the fact that this is the oldest antibiotic from the group of macrolides that has been used in medical treatment (Markiewicz and Kwiatkowski 2012). Disk-diffusion tests demonstrated that 9.1% of the tested *Staphylococcus* strains showed inducible mechanism of resistance to macrolides, lincosamides and streptogramins B. In the case of constitutive mechanism, it was 14.5% of the tested strains. The results presented by Lina et al. (17) show that among coagulase-negative staphylococci the inducible mechanism was detected in 28.7% of strains, while constitutive one—in 36.6%. On the other hand, Castro-Alarcon et al. (2011) reported that 47% of *S. epidermidis* strains tested in their study showed constitutive mechanism while inducible one was detected in 8% of strains. They also determined that 16% of strains were characterized by the MS_B_ type of resistance, while 11% showed the resistance to lincosamides. In contrast, in our study the MS_B_ type of resistance was found in as many as 38% of isolates.

Among the analyzed group of genes conferring the MLS_B_ type of resistance were genes determining the resistance to macrolides, i.e., *mph(C)* and *msr(A1)*—they were detected in more than half of the tested strains. Similar observations were reported by Lenart-Boroń et al. (2016) when analyzing the material of hospital-derived staphylococci with *mph(C)* gene observed in 62% of isolates and *msr(A1)*—in 89%. It should, however, be noted that there were also some significant differences in the occurrence of other resistance-determining genes between the hospital strains tested by Lenart-Boroń et al. (2016) and staphylococci analyzed in this study. Lenart-Boroń et al. (2016) detected *erm(B1)* (55% of isolates) and *mph(A)* (9% of isolates) genes, whereas they were not observed in our study. These differences could result from different origin of the analyzed strains, since hospital isolates can acquire various genes conferring antimicrobial resistance much more frequently (Murray 2007; Castro-Alarcon et al. 2011). The isolates analyzed in this study had a maximum of 7 resistance-determining genes at the same time, and some of them did not have any of the searched genes. Most often, individual strains had 4 resistance genes; however, having numerous resistance genes is not always beneficial for bacterial cells. When lacking antimicrobial pressure, the expression of genes determining such resistance is a significant effort for the cell metabolism and may reduce the growth of microorganisms (Marciniak 2008).

The increasingly detected, worrying process of antimicrobial resistance in *Staphylococcus* spp., which has been observed for many years, must be prevented. Currently, a number of educational activities are undertaken both in Poland and in other parts of the world, aiming at reducing abuse and misuse of antibiotics. This problem ceased to be the subject of interest of scientific community only, but it has become global and requires urgent intervention. This should be aimed at raising awareness of professionals, managers and public opinion about consequences and risks associated with uncontrolled use of antibacterial agents and methods to prevent drug resistance. Due to their global range, these issues have been considered as a priority in public health by a number of organizations and agencies around the world, e.g., by: the World Health Organization, the European Parliament, European Centre for Disease Prevention and Control, Centre for Diseases Control and Prevention, or Food and Drug Administration (CDC 2002; The European Commission 2001). The aim of all measures taken is to limit the uncontrolled overuse of antibiotics in medical and non-medical areas, prevent an increase in drug resistance and implement various programs in this regard. In addition, it is planned to develop a comprehensive system designed to stop the loss of antibiotic effectiveness in the treatment of infectious diseases as a result of the emergence and spreading of drug-resistant microorganisms, and to implement the principles of rational use of antibiotics.

## Conclusions

The group of analyzed airborne staphylococci isolated from flats in Kraków was dominated by *S. hominis* and *S. warneri* species. In this group, the resistance to erythromycin and tetracycline was most frequently detected; however, 31% of strains were susceptible to all tested antimicrobial agents including cefoxitin, ciprofloxacin, erythromycin, gentamycin, clindamycin, tetracycline and trimethoprim/sulfamethoxazole. A large variation was found in the prevalence and composition of antibiotic resistance-determining genes among the tested strains. The most common antibiotic resistance genes were *mphC* and *msrA1*, conferring the resistance to macrolides. The resulting antimicrobial resistance profile gives grounds to conclude that in the case of staphylococci infection, the use of antibiotics from basic antibiogram will be effective in therapy.
